# Draft Genome Sequence of Pseudomonas aeruginosa AS_23, Isolated from Petroleum-Contaminated Soil in Delta State, Nigeria

**DOI:** 10.1128/mra.00774-22

**Published:** 2022-09-12

**Authors:** Richard J. Kutshik, Grace A. Okenmor, Ishaya Y. Longdet

**Affiliations:** a Department of Biochemistry, University of Jos, Jos, Nigeria; University of Arizona

## Abstract

Pseudomonas aeruginosa AS_23 was isolated from petroleum-contaminated soil in Nigeria. Here, we present the draft genome sequence, consisting of an 6,760,157-bp genome with 6,348 protein-coding sequences, 6,253 functionally assigned genes, and 61 RNA genes. Average nucleotide identity-based whole-genome comparisons suggest that this is a new strain of Pseudomonas aeruginosa.

## ANNOUNCEMENT

Pseudomonas aeruginosa is ubiquitous, and the strains have a suitable genetic repertoire for this lifestyle (1, 2). Their broad spectrum of habitats reflects a high potential for adaptation to diverse environmental conditions and the ability to exploit diverse nutrient sources (3). Here, we report the draft genome sequence of Pseudomonas aeruginosa AS_23 from Nigeria. The organism was isolated from soil (pH 5.53) that had been collected (to a depth of 5 cm, using a soil auger) at a petroleum mining site in Delta State, Nigeria (5.35N, 6.10E), put into sterile sample bottles, kept in an airtight zipped bag, and transported to the laboratory. The soil was dried for 5 days, and 1 g was suspended in 9 mL sterile water and vigorously shaken (4). From this stock, serial dilutions (10^−1^ to 10^−10^) were prepared. An aliquot (0.1 mL) from each dilution was plated on a sterile nutrient agar plate and incubated at 37°C for 48 h. Bacterial growth was recorded only from the 10^−1^, 10^−2^, 10^−3^, and 10^−4^ dilutions. The plate with the 10^−4^ dilution was selected for the study. Based on morphological observations, four colonies were identified, and each was plated on a nutrient agar plate by streaking and incubated at 37°C for 24 h. To obtain pure colonies, each colony was streaked on two other nutrient agar plates and incubated at 37°C for 24 h in succession. The isolates were grown overnight in nutrient broth at 37°C. A 2-mL portion was centrifuged at 14,000 × *g* for 3 min, and the supernatant was discarded. This procedure was repeated with a fresh 2-mL portion. To wash the cell pellets, 500 μl of phosphate-buffered saline was added, and the mixture was centrifuged at 14,000 × *g* for 3 min. The washing was repeated.

The genomic DNA was extracted using a ZymoBIOMICS DNA miniprep kit. Default parameters were used for all software unless otherwise specified. DNA libraries were prepared using the Nextera XT kit (Illumina) and the Nextera Index kit (Illumina) and fragmented using Illumina Nextera XT fragmentation enzyme. Twelve cycles of PCR were performed to construct DNA libraries, which were purified using AMPure magnetic beads, eluted in Qiagen EB buffer, and quantified using a Qubit 4 fluorometer and the Qubit double-stranded DNA (dsDNA) high-sensitivity (HS) assay kit. Genome sequencing was performed with the Illumina HiSeq X Ten platform (2 × 150-bp paired-end sequencing). Trimming of raw single-end reads was done using BBDuk (v37.78) (5) with a read quality trimming parameter of 20. The trimmed fastq reads (with 3.01× depth [in millions]) were assembled using SPAdes (v3.13.0) (6), giving 129 contigs with a mean size of 52,404.32 bp, an *N*_50_ value of 114,588 bp, a total assembled size of 6,760,157 bp, genome coverage of 66×, and a GC content of 66.07%. The completeness evaluation using CheckM (v1.1.2) (7) estimated that the genome was 99.34% complete, with 2% contamination. The genome was annotated using PGAP (v6.1) (8), which identified 6,348 protein-coding sequences, 6,253 proteins with functional assignments, 95 pseudogenes, 3 rRNA operons, 54 tRNA genes, 4 noncoding RNA genes, and 2 CRISPR arrays. The whole-genome computed average nucleotide identity using MUMmer v4 (9) indicated about 99% similarity between Pseudomonas aeruginosa AS_23 and Pseudomonas aeruginosa UCBPP-PA14 (GenBank assembly accession number GCF_000014625.1), Pseudomonas aeruginosa B136-33 (GenBank assembly accession number GCF_000359505.1), and Pseudomonas aeruginosa VRFPA04 (GenBank assembly accession number GCF_000473745.2).

### Data availability.

This genome has been deposited in DDBJ/EMBL/GenBank under accession number JANAWV000000000. The version described in this paper is version JANAWV010000000. The raw reads were deposited in the Sequence Read Archive (SRA) under BioProject accession number PRJNA815837 and BioSample accession number SAMN29499814.
